# The Relationship Between Percentage Weight Loss and World Health Organization-Five Wellbeing Index (WHO-5) in Patients Having Bariatric Surgery

**DOI:** 10.1007/s11695-022-06010-2

**Published:** 2022-03-19

**Authors:** Roshaida Abdul Wahab, Heshma Al-Ruwaily, Therese Coleman, Helen Heneghan, Karl Neff, Carel W. le Roux, Finian Fallon

**Affiliations:** 1 Diabetes Complications Research Centre, Conway Institute, University College Dublin, Belfield, Dublin 4, Ireland; 2 Obesity Complications Clinic, St Vincent’s Private Hospital, Merrion Road, Dublin, D04 NE02 Ireland

**Keywords:** Obesity, Depression, Bariatric surgery, WHO-5 index

## Abstract

**Purpose:**

The association between bariatric surgery outcome and depression remains controversial. Many patients with depression are not offered bariatric surgery due to concerns that they may have suboptimal outcomes. The aim of this study was to investigate the relationship between baseline World Health Organization-Five Wellbeing Index (WHO-5) and percentage total weight loss (%TWL) in patients after bariatric surgery.

**Materials and Methods:**

All patients were routinely reviewed by the psychologist and screened with WHO-5. The consultation occurred 3.5 ± 1.6 months before bariatric surgery. Body weight was recorded before and 1 year after surgery. A total of 45 out of 71 (63.3%) patients with complete WHO-5 data were included in the study. Data analysis was carried out with IBM SPSS Statistics (version 27) to determine the correlation between baseline WHO-5 and %TWL in patients having bariatric surgery.

**Results:**

Overall, 11 males and 34 females were involved with mean age of 47.5 ± 11.5 and BMI of 46.2 ± 5.5 kg/m^2^. The %TWL between pre- and 1-year post-surgery was 30.0 ± 8.3% and the WHO-5 Wellbeing Index mean score was 56.5 ± 16.8. We found no correlation between %TWL and the WHO-5 Wellbeing Index (*r* = 0.032, *p* = 0.83).

**Conclusion:**

There was no correlation between the baseline WHO-5 Wellbeing Index and %TWL 1-year post-bariatric surgery. Patients with low mood or depression need to be assessed and offered appropriate treatment but should not be excluded from bariatric surgery only based on their mood.

**Graphical Abstract:**

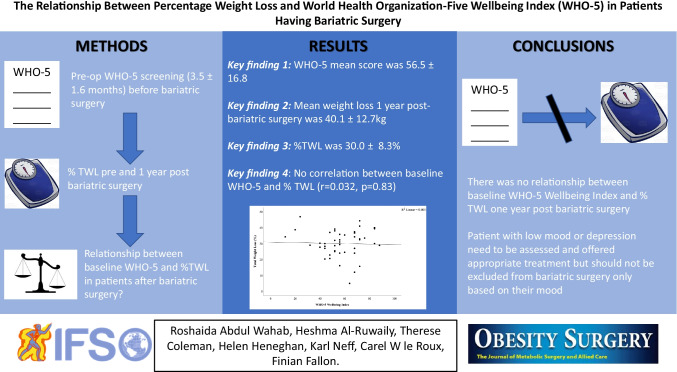

## Introduction/Purpose

Obesity is defined as a complex chronic disease characterized by excessive adipose tissue which causes a deterioration in health [1, 2]. There are numerous complications from obesity such as obstructive sleep apnea, non-alcoholic fatty liver disease, osteoarthritis, reduction in fertility [3], and also cardiometabolic diseases and cancer which reduces life expectancy [4, 5]. However, the impact on mental health cannot be underestimated [6]. There is a bidirectional association between obesity and depression [7–9]. This means that the presence of one condition increases the risk of developing the other and vice versa. Evidence suggests shared biological mechanisms linking obesity and depression such as overlapping genetic bases [10], hyperactivation of the hypothalamic–pituitary–adrenal axis [11, 12], and immuno-inflammatory activation which altogether alter the brain activity for mood and food [13].

Appropriate management of obesity needs to prevent the complications and improve physical and mental wellbeing. Such strategies may include medical nutrition, exercise, psychotherapy, pharmacotherapy, and bariatric surgery [14]. Bariatric surgery remains an effective treatment to treat obesity-related complications, maintain long-term weight loss, and improve quality of life [15, 16]. Evidence showed that bariatric surgery is associated with short-term [17] and long-term reduction of depressive symptoms up to 10 years post-operatively [18]. It is postulated that weight loss as a result of the surgery may lower the levels of pro-inflammatory cytokines which may be partly responsible for the depression [19].

The association between depression and obesity [20–22] is across gender and racial groups [23]. However, it is controversial whether there is a relationship between depression and bariatric surgery outcomes [24]. Several studies showed that bariatric surgery is beneficial for depression [25–28]. However, there is also evidence that patients might worsen following surgery [26, 29, 30]. Pre-operative depression also does not appear to be associated with the outcome of surgery [31]. Many patients with depression are not offered bariatric surgery because of the concerns that they may have suboptimal outcomes [32]. The World Health Organization-Five Wellbeing Index (WHO-5), a questionnaire to screen for depression, is a useful screening tool to evaluate patient’s current mental wellbeing pre-surgery. It is a generic wellbeing scale without any diagnostic specificity but has been shown to be beneficial across many study fields [33]. More than half of patients with obesity may present with psychiatric illness such as depression [34]. Patients after bariatric surgery are also at an increased risk of suicide compared to the background population [35]. Thus, a multidisciplinary team approach which includes clinical psychologists and psychiatrists plays an important role in optimizing patient care. Our hypothesis was that there is no correlation between baseline WHO-5 and percentage total weight loss after 1 year. Therefore, the aim of this study was to examine the relationship between baseline World Health Organization-Five Wellbeing Index (WHO-5) and percentage weight loss in patients after bariatric surgery.

## Materials and Methods

Approval for the evaluation of prospectively collected data was obtained from the hospital’s clinical audit and ethics committee (Clinical Audit Reference Number 3180). Patients who underwent bariatric surgery between 8 November 2018 to 10 August 2020 were included in the study. As part of the standard of care, patients were reviewed by the in-house psychologist and screened with WHO-5 before surgery. Body weight was recorded pre- and 1 year after surgery. The %TWL between pre- and 1-year post-intervention were calculated using Microsoft Excel. Data analysis was carried out with IBM SPSS Statistics software (version 27) to determine the correlation between %TWL and World Health Organization-Five Wellbeing Index (WHO-5) in patients having bariatric surgery. Both parameters were normally distributed and Pearson correlation was utilized to measure the linear association between the two variables.

The WHO-5 Wellbeing Index is a short questionnaire performed during patients’ pre-operative assessment with the clinical psychologist. Five statements require the respondents to rate their opinions on a scale of 0 to 5 (Table 1). To obtain the final score, the total raw score is then multiplied by 4. A total of 100 score defines the best respondents’ wellbeing and zero as the worst. A low score can be indicative of depression (≤ 50/100), with clinical depression indicated for scores of ≤ 28/100 [36].

**Table 1:**
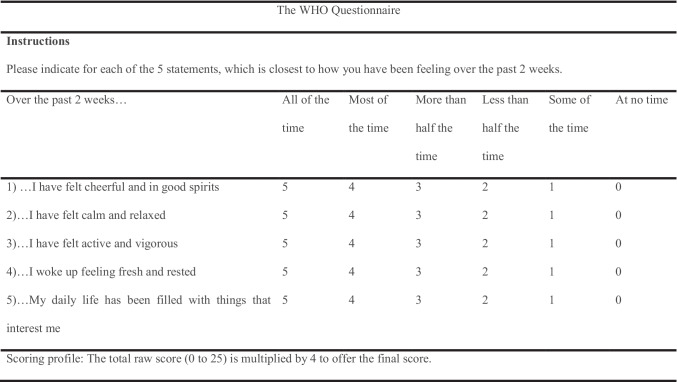
Example of the WHO-5 Wellbeing Index Questionnaire

## Results

A summary of baseline characteristics of the patients for which the data was collected is represented in Table 2. Overall, 45 out of 71 patients (63.3%) who had bariatric surgery were found to have baseline WHO-5 recorded (*n* = 11 males, *n* = 34 females) despite the clinic protocol stating all patients should routinely have WHO-5 score pre-operatively as part of usual care. The mean age and BMI were 47.5 ± 11.5 years old and 46.2 ± 5.5 kg/m^2^ respectively. The WHO-5 questionnaire was conducted on average 3.5 ± 1.6 months prior to bariatric surgery.

**Table 2:** Population baseline characteristics

Characteristics	Participants
*N*	45 (11 males, 34 females)
Age, y	47.5 ± 11.5
Sex (male %)	11 (24.4%)
Height, m	1.7 ± 0.1
Weight, kg	133.6 ± 25.0
BMI, kg/m^2^	46.2 ± 5.5

Mean ± SD (all such value)

**Fig. 1:**
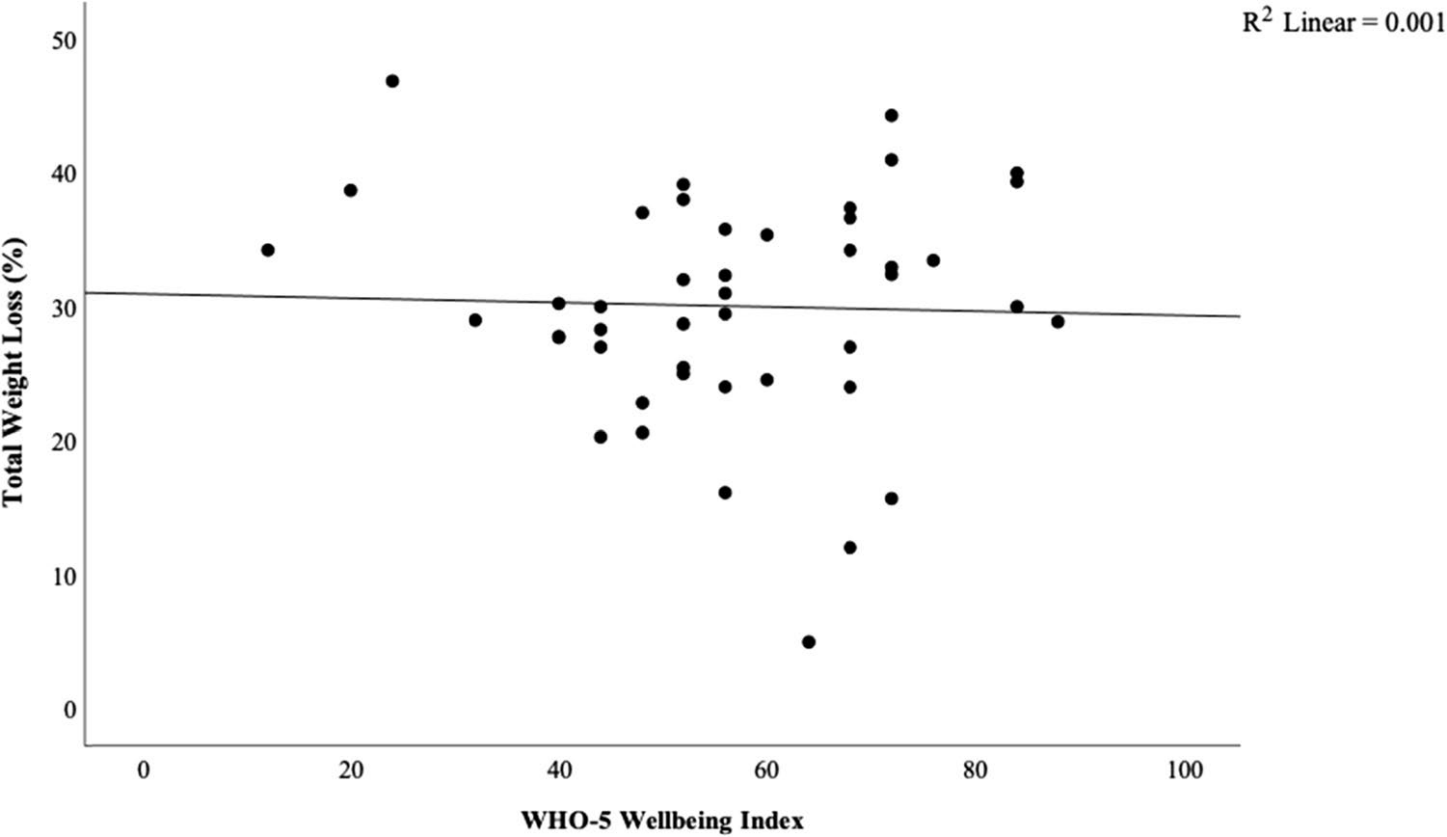
Correlation between percentage total weight loss 1-year post-bariatric surgery and the WHO-5 Wellbeing Index indicating no association (*r* = 0.032, *p* = 0.83)

The mean and SD pre-op weight was 133.6 ± 25.0 kg and the mean weight loss between pre and 1 year after the bariatric surgery was 40.1 ± 12.7 kg. The overall %TWL 1-year post-surgery was 30.0 ± 8.3%. The WHO-5 Wellbeing Index mean score was 56.5 ± 16.8. A total of 12 patients had WHO-5 wellbeing score < 50. Two patients had further psychiatric evaluation, another two were advised to continue psychotherapy, and five patients were asked to complete counselling prior to surgical approval. There were three out of 12 patients who did not require any additional mental health input, because they had sufficient support from their general practitioners or local mental health teams and thus were deemed suitable for surgery. Our investigation demonstrated that there was no significant correlation between %TWL and the WHO-5 Wellbeing Index (*r* = 0.032, *p* = 0.83) as shown in Fig. 1.

## Conclusion

There was no correlation between the WHO-5 Wellbeing Index at baseline and the %TWL after bariatric surgery. The WHO-5 Wellbeing Index is one of the most widely used and reliable psychometric measurements worldwide [37]. Other depression screening tools for bariatric surgery candidates include the Beck Depression Inventory (BDI), Hospital Anxiety Depression Scale (HADS), Patient Health Questionnaire (PHQ-9), and Mini International Neuropsychiatric Interview (MINI) [38, 39]. The WHO-5 Wellbeing index was developed to assess mental wellbeing but can be employed as a screening tool for depression as it demonstrates a high sensitivity for this disorder. For example, using a cut-off score of ≤ 50 to screen for clinical depression, several studies revealed a sensitivity for Diagnostic and Statistical Manual of Mental Disorders, fourth edition (DSM-IV) depression ranging from 0.77 to 0.96 [40–42] and a specificity between 0.65 and 0.89 [40, 43, 44]. Furthermore, the WHO-5 Wellbeing Index is a short, non-invasive questionnaire which is applicable to various study fields such as endocrinology [45], cardiology [46], neurology [44], and psychology [47], and can be used across different age groups [48, 49]. Therefore, it is a useful screening tool in a bariatric clinic to examine patient’s current mental wellbeing pre-surgery.

Previous studies indicate a conflicting relationship between depression and bariatric surgery outcomes. For example, several studies suggested that patients with depression are unsuitable for surgery [32, 50, 51]. Bauchowitz et al. conducted a survey involving 188 bariatric surgery programs to examine psychological assessment practices for bariatric surgery candidates in the USA [32] and 95% of the programs considered current symptom of depression as a possible contraindication to surgery. Another study also found a negative relationship between depression and bariatric surgery outcomes [51]. Kalarchian et al. showed that patients diagnosed with Axis I clinical disorder, e.g., mood or anxiety disorders, using Structured Clinical Interview for the DSM-IV were associated with poorer weight outcomes 6 months post-operatively compared to the subjects without Axis I clinical disorder. However, this study examined outcomes 6-month post-surgery and they did not report nadir weight which usually occurs later [15]. Other studies have suggested that patients with depression performed better after surgery [52–54]. In a survey conducted on patients after bariatric surgery (*n* = 1117), Odom et al. showed that higher BDI scores were correlated with a lower risk of weight regain 1-year post-surgery [54]. Another study showed that patients with pre-operative depression (measured by BDI) had more post-operation weight loss after 1 year [53]. However, a number of studies found no correlation between pre-operative depression versus weight loss in patients with bariatric surgery [31, 55, 56]. Powers et al. investigated 131 patients at 2 years and 5.7 years post-operatively with known psychiatric assessments prior to surgery [55]. They detected no relationship between presurgical psychiatric disorder and weight loss at follow-up. In a study by Fuchs and colleagues, the outcome of bariatric surgery (laparoscopic sleeve gastrectomy and laparoscopic adjustable gastric banding) was examined in patients with pre-operative psychological evaluation (*n* = 590) [31]. The study observed that there was no difference in percentage excess weight loss (%EWL) and psychiatric disorders including depression at 1-year post-surgery (*p* = 0.76). In line with these studies, Semanscin-Doerr and colleagues also found no difference between the presence and absence of lifetime history of depression and %EWL [56]. Our result was in line with these studies.

The strength of our study was that the baseline WHO-5 Wellbeing Index was conducted by the same clinical psychologist, therefore, avoiding a potential bias that may occur in self-reporting method such as patients underreporting of symptoms [57]. The WHO-5 is an easy tool which can be readily adopted in any bariatric practice and to our knowledge, there is no published data examining the relationship between WHO-5 Index and %TWL in patients with bariatric surgery. The limitation of the current study includes a small sample size (*n* = 45) and only 1-year post-surgical follow-up, but the data is very clear suggesting a larger group size with longer follow-up may not provide different outcomes. In addition, the cohort of patients that we identified may have affected the data. Moreover, it was beyond the remit of this study to compare the outcomes of patients with low WHO-5 scores who received or did not receive psychological or psychiatric treatment because all those patients in our cohort were offered treatments as part of their multidisciplinary obesity care.

In conclusion, we found that there was no correlation between the baseline WHO-5 Wellbeing Index and %TWL 1-year post-bariatric surgery. Patients with low mood or depression need to be assessed and offered appropriate treatment but should not be excluded from bariatric surgery only based on their mood.
